# Adventitial Alterations Are the Main Features in Pulmonary Artery Remodeling due to Long-Term Chronic Intermittent Hypobaric Hypoxia in Rats

**DOI:** 10.1155/2015/169841

**Published:** 2015-02-08

**Authors:** Julio Brito, Patricia Siques, Silvia M. Arribas, Angel L. López de Pablo, M. Carmen González, Nelson Naveas, Karem Arriaza, Karen Flores, Fabiola León-Velarde, Ruth Pulido, Stefany Ordenes, M. Rosario López

**Affiliations:** ^1^Institute of Health Studies, Universidad Arturo Prat, Avenue Arturo Prat 2120, 11100939 Iquique, Chile; ^2^Department of Physiology, Faculty of Medicine, Universidad Autónoma de Madrid, c/Arzobispo Morcillo 2, 28029 Madrid, Spain; ^3^Department of Biological and Physiological Sciences, Faculty of Sciences and Philosophy/IIA, Universidad Peruana Cayetano Heredia, Avenue Honorario Delgado 430, Urb. Ingenieria, Distrito, Lima 31, Peru; ^4^Department of Preventive Medicine and Public Health, Faculty of Medicine, Universidad Autónoma de Madrid, c/Arzobispo Morcillo 2, 28029 Madrid, Spain

## Abstract

Long-term chronic intermittent exposure to altitude hypoxia is a labor phenomenon requiring further research. Using a rat model, we examined whether this type of exposure differed from chronic exposure in terms of pulmonary artery remodeling and other features. Rats were subjected to chronic hypoxia (CH, *n* = 9) and long-term intermittent hypoxia (CIH2x2; 2 days of hypoxia/2 days of normoxia, *n* = 10) in a chamber (428 Torr, 4,600 m of altitude) for 46 days and compared to rats under normoxia (NX, *n* = 10). Body weight, hematocrit, and right ventricle ratio were measured. Pulmonary artery remodeling was assessed using confocal microscopy of tissues stained with a nuclear dye (DAPI) and CD11b antibody. Both hypoxic conditions exhibited increased hematocrit and hypertrophy of the right ventricle, tunica adventitia, and tunica media, with no changes in lumen size. The medial hypertrophy area (larger in CH) depicted a significant increase in smooth muscle cell number. Additionally, CIH2x2 increased the adventitial hypertrophy area, with an increased cellularity and a larger prevalence of clustered inflammatory cells. In conclusion, CIH2x2 elicits milder effects on pulmonary artery medial layer muscularization and subsequent right ventricular hypertrophy than CH. However, CIH2x2 induces greater and characteristic alterations of the adventitial layer.

## 1. Introduction

There are several pathophysiological conditions, including exposure to high altitudes that can impair alveolar oxygen availability and cause hypoxic pulmonary vasoconstriction and an increase in pulmonary artery (PA) pressure. Consequently, there are PA structural changes, that is, remodeling of the pulmonary vascular tree, that further contribute to increased PA tone, pulmonary hypertension, and right ventricle hypertrophy (RVH) [1–3].

In lowlanders moving to high altitudes, the mechanism of high altitude pulmonary hypertension (HAPH) involves sustained pulmonary artery vasoconstriction and remodeling, whereas in natives born at a high altitude, the mechanism of HAPH involves exaggeration of the remaining remodeling from the fetal state [4]. Furthermore, natives and sea level newcomers chronically living at high altitudes have higher PA pressure and greater right ventricle mass than lowlanders [4]. However, the prevalence of the full clinical presentation of HAPH in these individuals is only 10–15% [5].

As a consequence of the recent settlement of mines and other activities at high altitudes (greater than 3,000 m) in Chile and other parts of the world, workers are exposed to longer durations of chronic intermittent hypoxia (CIH) than with any other type of altitude exposure. Over the course of many years, these workers repeatedly ascend to altitudes of 3,800 to 4,800 m to work in shifts for an average of 7–14 days and then return to rest at sea level for equal periods of time [6]. This condition, called the “Chilean miners' model of intermittent exposure,” certainly differs from obstructive sleep apnea (OSA) or other models of hypoxia [7]. Currently, there are more than 65,000 people under this labor condition in Chile alone, and the estimated rates of HAPH and RVH are 4% and 12%, respectively [8].

Long-term CIH is a rather new type of exposure to biological conditioning, and studies of the alterations in the pulmonary vasculature in this biological condition have been limited. The addition of more information is of the utmost importance. Animal models have played a key role in the study of the mechanisms implicated in HAPH. These animal models have developed pulmonary hypertension and RVH under chronic hypobaric hypoxia [9–11]. Some of these studies have also demonstrated that pulmonary hypertension was associated with functional alterations and PA structural alterations, that is, vascular remodeling [12]. Several studies have indicated that the adventitial layer played a major role in the process of pulmonary vascular remodeling under CH, including the remodeling of large vessels (conducting or elastic Pas) [12]. Much information about the effects of long-term CIH on the morphology of pulmonary circulation under these specific conditions has been lacking in humans and animals, notwithstanding the large body of literature on OSA and several short hypoxic regimes.

We previously observed an alteration in NO–O_2_ balance [13]. Therefore, this research characterized remodeling and alterations in the PA in a rat model of long-term CIH by examining changes in gross structure, cellular distribution, and content, to determine whether long-term CIH presented the same structural changes that occur under chronic hypoxic (CH). We compared these changes in rats under three conditions: CIH, CH, and normoxia (NX). We used confocal microscopy to study vascular remodeling, which offers several advantages, including the ability to detect cellular alterations in the number and distribution of infrequent cellular events, such as anomalous cells [14, 15]. To the best of our knowledge, this particular characterization of changes caused by long-term CIH has not been attempted previously, and new data would provide a better understanding of these phenomena.

## 2. Methods

### 2.1. Animal Model

A total of 30 adult, 3-month-old Wistar rats were used in this study. We used only male rats to avoid secondary changes related to hormonal influences. Geographical altitude (hypobaric hypoxia) was simulated in a hypobaric chamber at 428 Torr, equivalent to an altitude of 4,600 m, in the Universidad Arturo Prat facilities. The rats were randomly assigned to one of the following groups over a period of 46 days:

- normoxia control group (NX, *n* = 10);
- long-term chronic intermittent hypoxia (CIH2x2; *n* = 10); or
- chronic hypoxia (CH; *n* = 9).

The animal model for long-term CIH exposure has been described previously [9, 16, 17]. This model involves 2 days of hypoxia and 2 days of normoxia to include at least one full circadian cycle and to resemble closely the exposure regimens of human miners working in shifts at high altitudes. The control group was placed in the same room at sea level (22 ± 2°C; 12 h light/dark cycle and humidity 35 ± 5%) under conditions comparable to the hypoxic groups. The animals were maintained in separate cages with food (20 g of pellets/day per rat) and water provided* ad libitum*. The water was provided in bottles that were specifically designed for pressure changes. Housekeeping and replacement of food and water was carried out every 2 days. At the end of the protocol, the animals were euthanized under anesthesia with an overdose of ketamine (8 mg, intraperitoneally). Only 29 rats were analyzed because one CH rat died of an unknown cause.

Standard veterinary care was administered during all of the experiments, following institutional protocols for the study of animals. The procedures were submitted to and approved by the Institutional Research Ethics Committee of Universidad Arturo Prat.

### 2.2. Weight and Hematocrit

All of the rats were weighed, and hematocrit (Ht, %) and hemoglobin (Hb, g/dL) were measured at baseline and at 14, 30, and 46 days. The animals' weights were measured using an Acculab V-1200 electronic balance (Chicago, Il, USA). A blood sample was taken from the tail, and hematocrit was measured using a microcentrifuge (Eppendorf AG, Hamburg, Germany). The hemoglobin concentration was measured using a Coulter Electronics Counter (Cell Dyn 3700, Abbott, Santa Clara, CA, USA).

### 2.3. Heart and PAs

The heart and the lungs were removed in a block for further dissection. The heart was excised immediately over the union of the ventricles to atria. The right ventricle was detached from the heart, leaving* in situ* the septum portion with the left ventricle. Both ventricles were weighed using an analytic balance (Acculab V-1200, Chicago, Il, USA). The ratio of the weight of the right ventricle to that of the left ventricle plus the septum was used to measure the grade of RVH [16, 18, 19]. The removed lungs were placed in a Petri dish. The PA tree was dissected, cleaned of adherent tissue, and flushed with saline solution. The 4th order branches were fixed in 4% paraformaldehyde for 60 min, followed by washing in a phosphate-buffered saline (PBS) solution and staining as follows.

### 2.4. Staining Protocols

To study the general structure and cellular organization in the PA wall, a longitudinal section and several ring sections were cut with a blade at the same anatomical location. The sections were stained with the nuclear dye 4′,6-diamidino-2-phenylindole (DAPI, Sigma, St. Louis, MO, USA; 1 : 500 from a 5 mg/mL stock) for 30 min at room temperature (RT) in darkness and were washed twice in PBS (15 min, RT). To detect the presence of inflammatory cells, while another segment was first incubated with the primary antibody CD11b (Millipore, Bellerica, MA, USA) (60 min, 1 : 200 in PBS at RT) and then washed with PBS (30 min, RT). Thereafter, the segments were incubated with the secondary antibody Alexa Fluor 488 goat anti-mouse IgG (Invitrogen, Madrid, Spain; 60 min, 1 : 200, RT), were washed with PBS for 30 min, and were incubated with DAPI as described above.

### 2.5. Confocal Microscopy Acquisition

Two to three rings were mounted on a slide equipped with a small well made with spacers. The well was filled with mounting medium (FluoroGuard, Bio-Rad, Hercules, CA, USA), and the specimens were covered with a cover glass. Similarly, the longitudinal sections were mounted, one with the adventitial side facing up and the other with the endothelial side facing up, and the sections were visualized with a Leica TCS SP2 confocal system (Leica Microsystems, Wetzlar, Germany) at the Universidad Autonoma de Madrid of Spain facilities.

Ring sections were visualized at 488 nm excitation/500–560 nm emission wavelengths to determine the lumen size and medial thickness and to detect the elastic lamella because elastin is autofluorescent at this wavelength (14). A 10x air objective was used to obtain single images of the complete ring to study lumen size. Several images were obtained to determine medial thickness using a 63x oil immersion objective at several locations of the arterial ring, where the four elastic lamellae were clearly visible. The adventitia is a diffuse layer, and therefore it could not be quantified from the ring sections. The adventitia was further analyzed from the longitudinal sections, as previously described [15, 20]. Briefly, serial, 1 *μ*m thick, optical sections were obtained from the first to the last visible adventitial cell (AC) in the artery using a 63x objective at zoom 2 at 405 nm excitation/410–475 nm emission wavelengths. From these images, the number of cells and their thickness were quantified, and the cell density was calculated. Similar images of the smooth muscle cell (SMC) layer were obtained from the other longitudinal sections, which were mounted with the endothelial side facing up to study the number and orientation of the SMCs.

The presence of inflammatory cells in the PA was detected using double staining with the nuclear dye DAPI (405 nm excitation/410–475 nm emission) to locate cells and the specific antibody CD11b (Biosciences, Temecula, CA, USA), which recognizes most activated macrophages and granulocytes, and using Alexa Fluor secondary antibody (Invitrogen, Carlsbad, CA, USA; 488 nm excitation/500–600 nm emission). Serial images were obtained at both wavelengths with a 63x objective at zoom 4 from the longitudinal sections, as described above.

### 2.6. Quantitative Analysis

The perimeter was measured from ring images obtained with the 10x objective using Metamorph Image analysis software (Universal Imaging Corporation, Marlow, UK), and the data were used to calculate the size of the internal diameter. Ring images obtained with a 63x objective were used to quantify medial thickness by measuring the distance between the external and internal elastic laminae (3–5 measurements per image) using Metamorph software. The adventitial thickness was quantified from the number of serial images captured from the stacks of serial images obtained from the longitudinal sections. Wall cross-sectional area was calculated based on the measurements of the internal diameter and medial and adventitial thickness. The numbers of adventitial cells (ACs) and SMCs were counted using Metamorph software in a known volume, which was calculated from the layer thickness and image area [20]. The number of inflammatory cells in the vascular wall was quantified in a similar manner, and the results are expressed per vessel volume.

### 2.7. Statistical Analysis

Experimental data were entered into a database and were analyzed using SPSS software, version 17.0 (SPSS, Inc., Chicago, IL, USA). The mean, standard deviation, standard error (SE), and confidence interval were calculated for each parameter. Normality was established using the Kolmogorov-Smirnov test. Statistical analysis of the differences across all testing conditions and artery remodeling were established using one-way analysis of variance (ANOVA) and less significant deviation (LSD)* post hoc* tests. To analyze differences over time, repeated measures ANOVA was performed. All of the variables were normally distributed. Statistical significance was established at a *P* value < 0.05.

## 3. Results

### 3.1. Hematocrit and Weight

There was a loss of weight in the hypoxic groups: CIH2x2 (206 ± 8.03 g), compared to NX (330 ± 13.5 g), and CH, which was greater (169 ± 3.6 g); *P* < 0.001.

**Figure 1:**
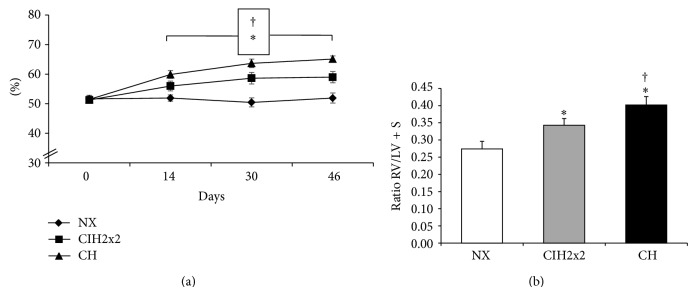
Hematocrit and right ventricular hypertrophy: (a) hematocrit (%) and (b) right ventricular hypertrophy (RVH), calculated by the right ventricle/left ventricle + septum (RV/LV + S) ratio, for all of rats exposed to CIH2x2, CH, and NX. Values are expressed as means ± SEs. Statistical comparisons were performed using repeated measures ANOVA for (a) and one-way ANOVA for (b); ^*^ *P* < 0.01  is exposed versus NX and ^†^ *P* < 0.05  CH versus CIH2x2.

Hematocrit was significantly elevated in CH (66% ± 1.1) and CIH (58% ± 1.8), compared to NX rats (51% ± 1.0), (*P* < 0.01). CIH hematocrit reached an intermediate value compared to CH (*P* < 0.01) (Figure 1(a)).

### 3.2. RVH Was Greater in CH Than CIH2x2

Right ventricular hypertrophy was observed in both exposed groups (*P* < 0.01). The hypertrophy was greater in the CH group than in the CIH2x2 group (*P* < 0.05), as shown in Figure 1(b).

### 3.3. Hypertrophic Remodeling of the PA Occurred in Both Hypoxic Groups without Changes in Internal Diameter

**Figure 2:**
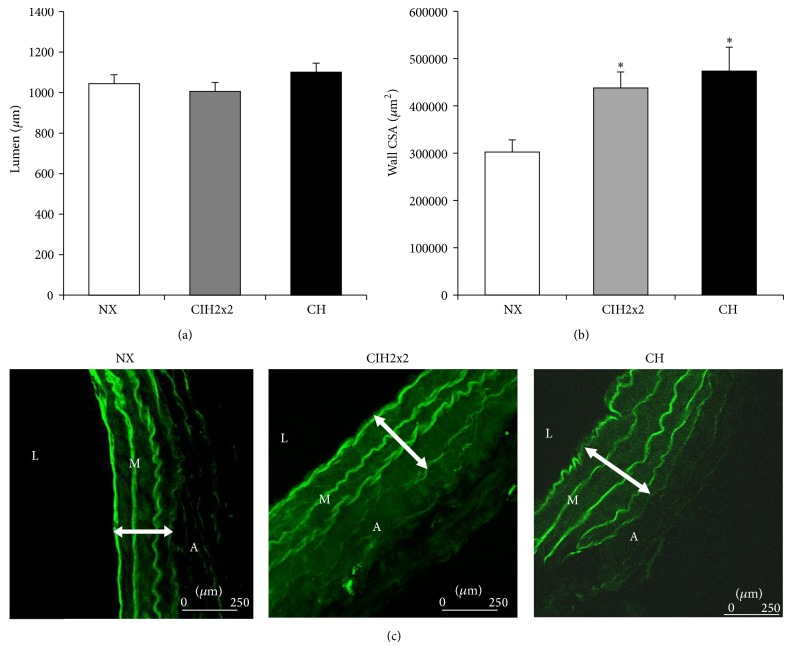
Pulmonary artery wall: (a) lumen: internal diameter in microns (*μ*m), (b) wall cross-sectional area (CSA) (*μ*m^2^), and (c) representative confocal images of the pulmonary artery wall of rats exposed to CIH2x2, CH, and NX obtained with a 63x objective. L: lumen, M: media layer, and A: adventitial layer. Values are means ± SEs. Statistical analysis was performed using one-way ANOVA. For wall CSA, ^*^ *P* < 0.01  is exposed versus NX.

There were no significant differences in arterial internal diameter, which was quantified from the perimeter size, between the control and exposed groups (Figure 2(a)). However, both exposed groups showed an increased wall cross-sectional area (CSA) of the PA compared to the NX group (*P* < 0.01), but there were no significant differences between the exposed groups (Figure 2(b)). We separately analyzed the contributions of the medial and adventitial layers to wall hypertrophy.

### 3.4. Features of Medial Remodeling in Both Hypoxic Groups

**Figure 3:**
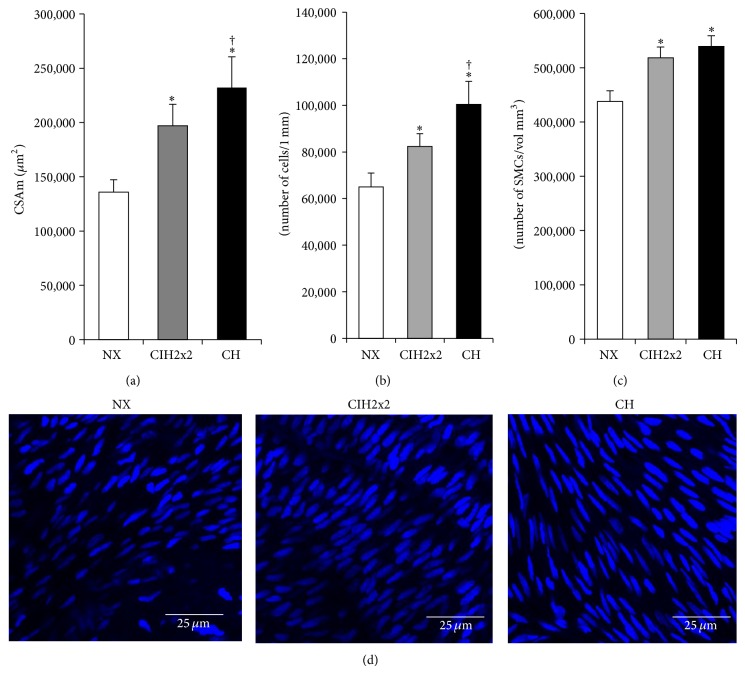
Media layer: (a) cross-sectional area (CSAm) of media layer in square microns (*μ*m^2^), (b) total number of smooth muscle cells (SMCs *μ*m/1 mm length artery), (c) total cell density (SMCs/layer volume mm^3^), and (d) representative confocal reconstructions obtained of the PA SMCs from 15 serial medial layer images of pulmonary arteries from rats exposed to CIH2x2, CH, and NX (63x objective zoom 2). Values are expressed as the means ± SEs. Statistical analysis was performed using one-way ANOVA. ^*^ *P* < 0.01  is exposed versus NX and ^†^ *P* < 0.05 CH versus CIH2x2.

The medial CSA was increased in both exposed groups compared to NX (*P* < 0.01), but it was increased by less in the CIH2x2 group than the CH group (*P* < 0.05) (Figure 3(a)). The media-to-lumen ratio (%) was also increased in both conditions, compared to the NX group (*P* < 0.01).

The total number of SMCs per 1 mm of artery was greater in both hypoxic conditions than in NX (*P* < 0.01), but fewer SMCs were observed in the CIH2x2 group than in the CH group (*P* < 0.05; Figure 3(b)). Additionally, there was a difference in the density of SMCs, which were measured as the numbers of SMCs per medial volume, between the exposed groups and the NX group (*P* < 0.01), but there were no significant differences between the exposed groups (Figure 3(c)). Arterial SMCs in the medial layer (defined as the region between the internal and external laminae) exhibited a typical spindle-like nuclear shape, and they were oriented perpendicularly to the flow direction, with no alterations in orientation [21], as shown in Figure 3(d).

### 3.5. Adventitial Remodeling Features in CIH Differed from Those in CH

**Figure 4:**
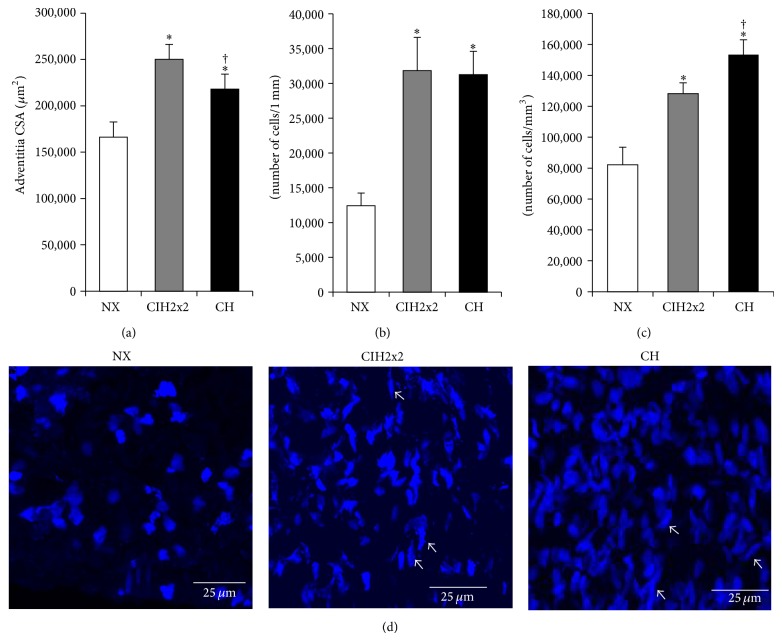
Adventitial layer: (a) adventitial CSA (*μ*m^2^). (b) Adventitial total cell number/1 mm of artery (N°. cells/mm) measured under a 10x objective. (c) Total density (cell number/layer volume; mm^3^) and (d) representative confocal reconstructions obtained from the adventitial layer of PAs (63x objective zoom) from rats exposed to CIH2x2, CH, and NX. Results are expressed as means ± SEs; statistical analysis was performed using one-way ANOVA. ^*^ *P* < 0.01  is exposed versus NX and ^†^ *P* < 0.05 for CH versus CIH2x2.

The adventitial CSA was significantly increased in both exposed groups compared to NX (*P* < 0.01), and it was greater in the CIH2x2 group than in the CH group (*P* < 0.05; Figure 4(a)). The number of ACs was strikingly increased in both exposed groups, and it was nearly threefold higher than in the NX group (*P* < 0.001; Figure 4(b)). The density of ACs was also increased (*P* < 0.01), and it was greater in the CH group than in the CIH2x2 group (*P* < 0.05; Figures 4(c) and 4(d)). We also observed cells with anomalous nuclear shapes that were more elongated and that resembled a spindle-like SMC shape in both hypoxic groups.

**Figure 5:**
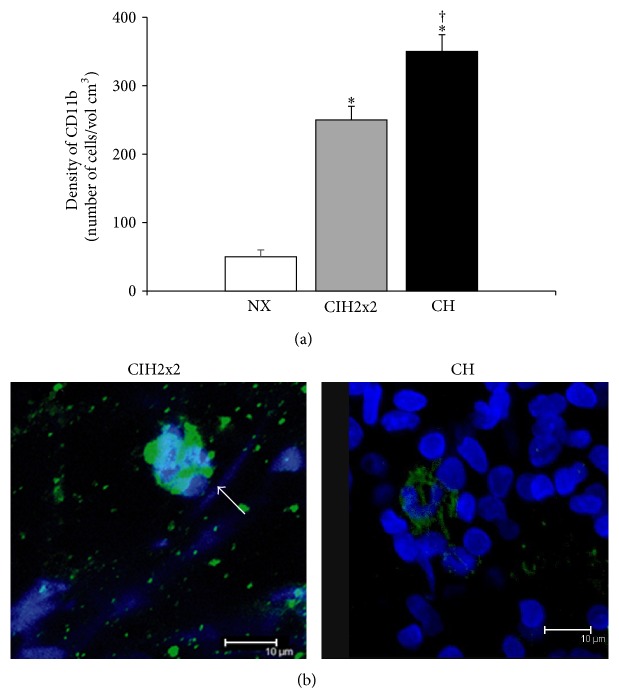
Adventitia and inflammatory cells: (a) density of adventitial cells positive for CD11b (expressed as N° of cells/cm^3^) and (b) representative confocal images obtained by reconstruction of the adventitia of arteries stained with the nuclear dye DAPI (405 nm excitation/410–475 nm emission) and the specific antibody CD11b and Alexa Fluor 488 secondary antibody (488 nm excitation/500–600 nm emission). Serial images of the adventitia of arteries from rats exposed to CIH2x2, CH, and NX were captured at both wavelengths with a 63x objective zoom. Results are expressed means ± SEs. Statistical analysis was performed using one-way ANOVA. ^*^ *P* < 0.001  is exposed versus NX and ^†^ *P* < 0.001 CH versus CIH2x2.

The number of CD11b-stained cells per volume in the adventitia increased in both hypoxic groups compared to the NX group (*P* < 0.001), and the cell density was greater in the CH group than in the CIH2x2 group (*P* < 0.001, Figure 5(a)). Interestingly, the inflammatory cells in the CIH2x2 group depicted the unique feature of gathering in clusters in 75% of observations, unlike cells in the CH group (Figure 5(b)).

## 4. Discussion

The main findings of the present study were that long-term CIH in rats led to vascular remodeling and RVH, as in CH, but the structural changes appeared to be milder. Both hypoxic conditions were accompanied by SMC hyperplasia, which might participate in the sustained elevated pulmonary pressure that increased the vascular tone and produced subsequent RVH. Most importantly, we found that the adventitial layer might have a greater and characteristically active role in the remodeling process under long-term intermittent exposure to hypoxia through proliferative and inflammatory processes that were not previously described for the current model.

### 4.1. Remodeling in Hypoxia

Remodeling has been a matter of concern in pulmonary hypertension induced by CH resulting from high altitudes, chronic obstructive pulmonary diseases, or other medical causes [4, 22]. Remodeling under CH has been characterized by an increased muscularization of the pulmonary vasculature, with an increase in medial thickness (mostly of small arteries), right heart hypertrophy, and angiogenesis [1, 23]. Additionally, there are adventitial thickening [24], migration and proliferation of SMCs, and an increase in extracellular matrix deposits along the entire wall [25]. These wall changes are correlated with PA pressure, and the basic structural changes in pulmonary vasculature are rather similar and are independent of the primary cause [11]. Our results in the 4th order PA from rats exposed to CH conditions were fairly similar to those of previous studies conducted in chronically exposed rats [11, 25], with the exception that we did not find a reduction of lumen size as previously reported [26]. We suggest that this difference was likely related to the larger artery size used in our study, compared to the small arteries (<200 m in diameter) used in previous reports. It is likely that larger vessels do not exhibit lumen encroachment but that pulmonary resistance arteries do, as observed in systemic hypertension [21]. Similar gross structural alterations were observed in this intermittent model.

With the aid of confocal microscopy, we analyzed the specific contribution of the adventitial and medial layers to the hypertrophy of the entire wall. Our results indicated that these layers contributed to different extents, depending on the hypoxia exposure regime, which was somewhat expected (i.e., greater total hypoxia dose over lower hypoxia dose). Therefore, there was a greater contribution of the medial layer in CH, which involved SMC hyperplasia and media enlargement, compared to intermittent exposure, in which there was also an increase in cell number but to a smaller extent. This phenomenon could explain the larger RVH observed in CH compared to CIH2x2, suggesting that CIH represents a milder form of pulmonary artery hypertension and subsequent remodeling. Notably, the observed enlargement in medial CSA and the increased number of SMCs in CH have been suggested to be a consequence to sustained hypoxic pulmonary vasoconstriction (HPV) [4, 27–29]. However, this enlargement could also be direct response to hypoxia, mediated by HIF-1. Consistent with this hypothesis,* in vitro* cellular experiments have shown that HIF-1 plays a pivotal role in pulmonary artery SMCs proliferation [30, 31].

The adventitia is an important layer that is involved in vascular remodeling, and this layer is increased in size in systemic hypertension models [15, 20] and during hypoxia and pulmonary hypertension [12]; however, there is no description of its changes under long-term CIH. We observed that CH and CIH2x2 were accompanied by important adventitial hypercellularity and increased volume, but adventitia enlargement was much greater in long-term CIH than in CH. It is well known that the adventitial compartment of the vessel wall suffers the earliest and most dramatic structural changes following hypoxic exposure in humans and in animal models [29], and this layer promoted the development of a PA-specific proinflammatory microenvironment [32]. There is a wide array of cells that have been described as being involved in adventitial wall changes under hypoxia, including resident adventitial fibroblasts in PA [33], which might undergo proliferation [34] and contribute to enlargement of the wall. Consistent with this latter hypothesis, we observed a variety of cells in the adventitial layer with an anomalous shape, resembling SMCs, which could be adventitial fibroblasts capable of differentiating into SMC-like cells, particularly myofibroblasts, as previously described [12].

Moreover, sustained hypoxia also promotes the development of an inflammatory process in the PA [32], and emerging evidence has suggested that circulating inflammatory and/or progenitor cells contribute significantly to the remodeling process [32, 35–37]. We confirmed the presence of inflammatory cells in the adventitia of both CH and CIH2x2 rats through staining with a CD11b antibody (which recognizes most activated macrophages and granulocytes) in a larger proportion than in CH rats. However, it is noteworthy that the quality of the cell distributions was different under the CH and CIH2x2 conditions. The inflammatory cells in CIH2x2 tended to be organized as clusters (75% of the observations), whereas they were isolated and had the characteristic shape of polymorphonucleated cells in CH. Nevertheless, it cannot be excluded that the difference in cellular counting between the two hypoxic groups could be due only to underestimation of the cells in CIH2x2 because the clustered cells made obtaining a proper count difficult. To the best of our knowledge, these morphological findings have not been previously reported in this condition. These cluster aggregation patterns of inflammatory cells in CIH2x2 could represent a different extent of inflammatory activity or a different adhesion status mediated by specific factors. Hypoxia is able to produce and/or release many inflammatory mediators, such as cytokines, from SMCs, fibroblasts, and platelets [30]. Interestingly, Ramirez et al. [38] found a significant increase in the inflammatory status and levels of inflammatory mediators in CH compared to CIH, which had a milder response. Investigating these possibilities is a matter for future research.

The increased thickness of the adventitia in CIH2x2 could not be explained by the increased cellularity alone because the density of the adventitial cells was smaller in CIH2x2 than CH. One likely explanation is an increase in extracellular matrix production, which is a common phenomenon in hypoxia, as a result of an upregulation of collagen, fibronectin, and tropoelastin mRNAs, followed by the subsequent deposition of these proteins [1] and enhancement of the turnover of matrix proteins under hypoxic conditions [39]. Another explanation for the greater increase in adventitial volume found in the current study might be related to a more specific inflammatory process associated with a probable reperfusion-injury phenomenon with subsequent edema. Congestion and edema have been previously described as characteristic in the same model used in our study [9]. The overall influence of this sort of reoxygenation and whether the mechanisms activated by hypoxia are turned off under normoxia have not been fully determined [40]. However, remodeling changes that occur at overtime at high altitudes are almost completely reversed after a period of time as sea level [41–43]. Whether there is a desensitization phenomenon in the responses to CIH is a controversial issue and possibly depends on the specific behavior of different organs in response to hypoxia [16, 41].

Further, the consequences of remodeling could also result in changes in impedance [44], resulting in a stiffening of more proximal PAs. In addition, a larger number of SMCs would exert a greater degree of vasoconstriction. Therefore, these changes would result in an increase in after-load pressure, an influence on right ventricle function, a remodeling of cardiomyocytes and fibroblasts, and ultimately RVH [45]. Moreover, the milder reduction of NO availability in long-term CIH compared to CH due to destruction by superoxide anions, previously found in this rat model [13], could be anther relevant mechanism to explain the milder degree of remodeling and subsequent RVH found in our study in long-term CIH. On the other hand, cardiac interstitial fibrosis, which could be the result of a profibrotic state mediated by HIF-1*α* [46], does not seem to play a significant role in this model of CIH, as demonstrated in a previous study [9].

**Figure 6:**
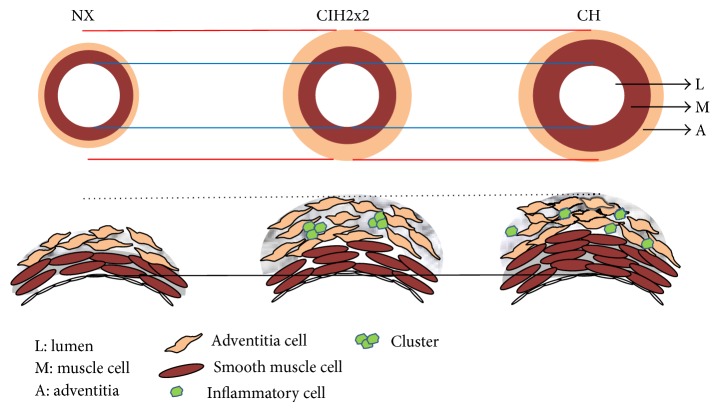
Schematic diagram showing the main changes observed in the PA of rats under CIH2x2, compared to CH and NX.

There were several limitations to consider to translate our results fully to clinical grounds. All rats develop RVH as a consequence of exposure to any type of hypoxia, whereas the most frequent CIH (OSA) in humans occurs in only 20–40% of patients [47]. What facets of RVH found are part of a “physiological phenomenon of high altitude” must be determined [4] versus what factors are truly an HAPH consequence. Current studies under way in our laboratory in humans (Brito et al., nonpublished data) have shown than more than 84% of the subjects in long-term CIH develop right ventricle enlargement. Additionally, whether our current rat model fully resembled the miners' shift conditions must be determined, although it has been validated previously [9, 13, 16]. A schematic diagram is provided showing the main changes observed in the PA of rats' under CIH2x2 compared to CH and NX (Figure 6).

## 5. Conclusions

In conclusion, our current results indicated that actual PA remodeling occurred in this emerging model of long-term CIH but with some specific characteristics not described before. The main morphological features seemed to be milder than in CH and can be summarized as follows: a greater increase in the adventitial layer, hypercellularity, and a particular pattern of inflammatory cell clustering. Future research is merited on this particular issue, which could help to predict and determine potential clinical consequences.

## Supplementary Material

Video shows a confocal live view of adventitial inflammatory cells stained with CD11b from rat's PA exposed to CIH depicting its particular cluster aggregation.
